# Biochemical functionality of magnetic particles as nanosensors: how far away are we to implement them into clinical practice?

**DOI:** 10.1186/s12951-019-0506-y

**Published:** 2019-05-31

**Authors:** Simon Doswald, Wendelin Jan Stark, Beatrice Beck-Schimmer

**Affiliations:** 1 Institute for Chemical and Bioengineering, ETH Zurich, Zurich, Switzerland; 2 Institute of Anesthesiology, University of Zurich and University Hospital Zurich, Raemistrasse 100, 8091 Zurich, Switzerland

**Keywords:** Magnetic nanoparticle, Nanosensors, Medical applications, Blood purification, Risk assessment

## Abstract

Magnetic nanosensors have become attractive instruments for the diagnosis and treatment of different diseases. They represent an efficient carrier system in drug delivery or in transporting contrast agents. For such purposes, magnetic nanosensors are used in vivo (intracorporeal application). To remove specific compounds from blood, magnetic nanosensors act as elimination system, which represents an extracorporeal approach. This review discusses principles, advantages and risks on recent advances in the field of magnetic nanosensors. First, synthesis methods for magnetic nanosensors and possibilities for enhancement of biocompatibility with different coating materials are addressed. Then, attention is devoted to clinical applications, in which nanosensors are or may be used as carrier- and elimination systems in the near future. Finally, risk considerations and possible effects of nanomaterials are discussed when working towards clinical applications with magnetic nanosensors.

## Background

Functionalization of magnetic nanoparticles (MNPs) for biomedical and clinical applications as nanosensors has received considerable attention in the past. Particularly in cancer diagnosis and treatment, magnetic nanosensors are widely explored as potential alternatives, either to identify less invasive diagnostic tools such as collection of cancer cells or tissue material or to specifically deliver medications within the tumor.

A magnetic nanosensor is a MNP functionalized with sensing moieties, which interact with proteins, DNA or cell surfaces. While many magnetic nanosensors are available, this review will exclusively focus on magnetic nanosensors designed to be used as diagnostic or therapeutic tools in medicine. Magnetic nanoparticles for general biomedical applications [1–5], or distinctive applications [6–11] are described elsewhere.

From a clinical perspective, it is important to make a distinction between magnetic nanosensors applied in vivo (intracorporeal approach) and magnetic nanosensors, which are used extracorporeally (extracorporeal approach). The distinction is important since different requirements for materials, properties and handling are needed. Concerning in vivo applications, drugs are attached to MNPs and carried to a specific location, a process called drug delivery. In another in vivo system, magnetic nanosensors as contrast agents are available to convert a biochemical information (e.g. tumor tissue) through radiation into an analytic signal (imaging of the tumor) in magnetic resonance imaging (MRI). In extracorporeal approaches such as blood purification, magnetic nanosensors are used to bind and collect specific biochemical or cellular entities that on the top allow for detailed analysis or diagnosis after recovery of the sensors. Blood purification with magnetic nanosensors can be considered similar to a dialysis device, whereas no sensor particles are allowed to reach the body after the purified blood is redirected to the patient [12].

When aiming towards magnetic nanosensors in clinical applications the material and synthesis are key for their functionality. Therefore, the following section will cover different synthesis pathways and functionalizations of MNPs. Subsequently, chances of applying nanosensors in different medical applications as well as risk considerations when using nanomaterials are discussed.

## Magnetic nanoparticles: material and synthesis

There are a number of ways to synthesize MNPs, which are described in detail by Schüth et al. [13]. As a brief overview, several techniques are used: (i) Co-precipitation, with which iron oxides are synthesized using aqueous salt solutions. Under inert atmosphere and through the addition of base, iron oxide is precipitated. (ii) Thermal decomposition, where organometallic precursors in high boiling solvents are decomposed by heating to either metal or metal-oxide nanoparticles. With this method, depending on solvent and precursor, very small particles (1–20 nm) are synthesized. (iii) Microemulsion: two immiscible liquids including a surfactant are used to form micelles. Within the micelles, the desired precursor is transformed to the corresponding nanoparticle. (iv) Hydrothermal synthesis: Li et al. [14] reported a liquid–solid-solution phase transfer synthesis pathway preparing various metal nanoparticles. Another method for preparing MNPs hydrothermally is the so called hot-injection technique [15]. It is a widely used method for the preparation of monodisperse nanoparticles and quantum dots. This approach involves the injection of a room temperature precursor solution, generally a metal-chloride or -methylate, into a hot high-boiling-point liquid. The high temperature hinders further nucleation. Therefore, it is possible to fabricate very size-uniform nanoparticles. (v) Flame synthesis: This is a synthesis route, which was first used to prepare oxide and non-oxide ceramics such as silica and pigmentary titania [16], followed by the production of various other oxide nanoparticles in pilot scale quantities [17]. Subsequently, this method has been adapted to produce also non-noble metal nanoparticles [18]. Due to their air instability, attributed to the small size of these metallic MNPs, they oxidize spontaneously. Therefore, such MNPs are not usable as possible candidates for magnetic nanosensors. By the addition of acetylene and subsequently modifying the flame synthesis conditions, however, metallic MNPs are stabilized with a carbon layer as shown using cobalt particles [19]. Carbon-coated metallic MNPs are air-, solvent- and in a wide range pH-stable [20]. Another significant advantage of carbon layers is the possibility to perform chemical modification in order to covalently bind functional groups. As a consequence, in the meantime many differently functionalized carbon-coated MNPs were designed [21–32].

With the described surface modification and functionalization carbon-coated MNPs became as interesting as the already widely explored superparamagnetic iron oxide nanoparticles (SPIONs) [33]. SPIONs are generally composed of γ-Fe_2_O_3_ or Fe_3_O_4_. In comparison to other metal and metal-oxide nanoparticles, SPIONs have the advantage of being compatible in a biological environment [34]. Also, they undergo biodegradation [34]. This has made SPIONs prominent candidates for in vivo applications. Biodegradation of SPIONs is dependent on coating and coating material as well as on size. Coating influences biodegradation due to partial hindered access to the metal-oxide core [35]. Concerning biodegradation, very small particles (< 20 nm) will be quickly eliminated in the body by the kidneys, whereas on the other hand large nanoparticles (> 200 nm) will be filtered in the liver and spleen [36]. These are important aspects when aiming at an in vivo application of the nanoparticles.

The main difference between carbon-coated MNPs and SPIONs, from a pure materials point of view, is the higher saturation magnetization of the former, which leads to a much faster separation of carbon-coated MNPs when applying a magnetic field. Additionally, SPIONs are superparamagnetic while carbon-coated MNPs are ferromagnetic [37]. This means that carbon-coated MNPs have a tendency to aggregate due to their permanent magnetization, which may impose a challenge when using bare carbon-coated MNPs for in vivo applications. However, aggregation may be hindered by surface modification of the carbon-coated MNPs to produce stable dispersions [38]. Furthermore, magnetic properties of nanoparticles can also be influenced by other factors than the material choice such as size of the particles, crystallinity, shape and composition [39]. This should be considered when tailoring nanoparticles towards specific properties and applications.

**Fig. 1:**
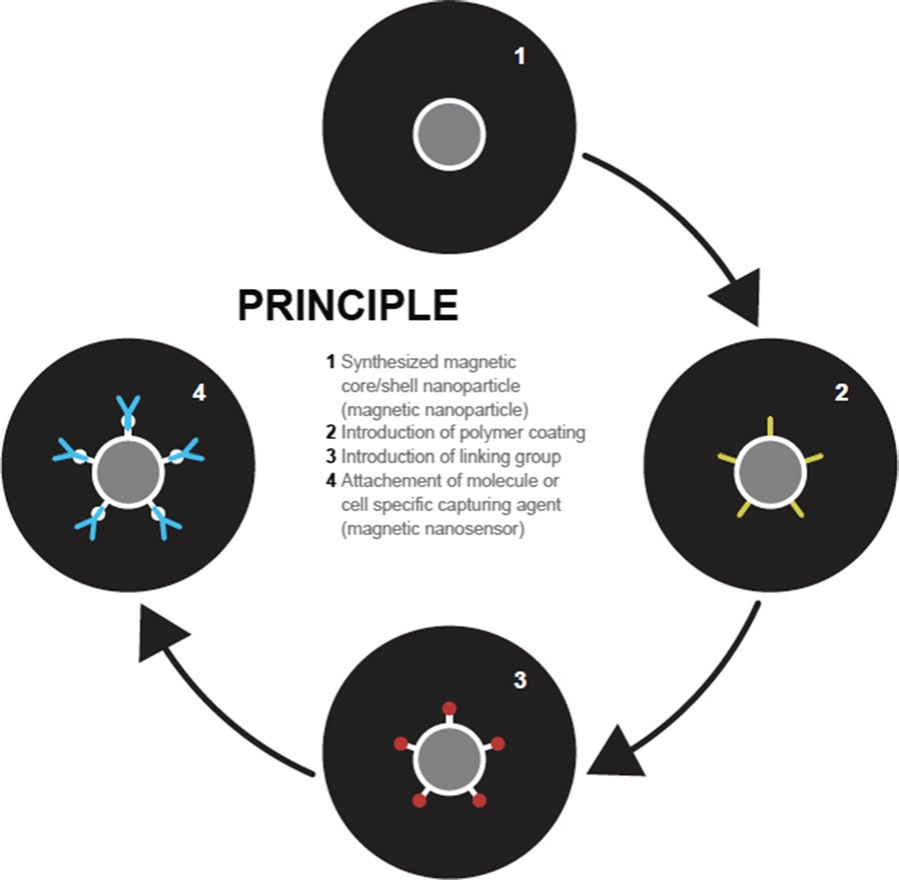
General synthesis procedure to fabricate a magnetic nanosensor from a magnetic nanoparticle. Production of the magnetic particle core entails providing the magnetic material and protecting it against dissolution or changes. Core shell geometries with silica, polymer or carbon coatings are the most frequently applied layers connecting the magnetic core with the biochemical functionality. After application of a linker, a specificity creating moiety must be attached. Suitable entities are antibodies, nucleic acids and other biomolecules

Physicochemical characteristics of the synthesized magnetic nanosensors determine biocompatibility. As a consequence, interactions with the biological milieu such as the blood have to be considered carefully. Blood is a complex liquid consisting of different molecular and cellular entities. Therefore, it is important to ensure that magnetic nanosensors do not interfere with blood in any other way than intended, nor should they induce any unwanted reactions such as inflammation. In general, a suitable coating with a polymer is sufficient to achieve biocompatibility before the sensing functionality is implemented (Fig. 1). A widely applied approach is dextran coating of nanosensors used as contrast agents for MRI [40–42]. Another possibility to coat MNPs with a polymer is the use of atomic transfer radical polymerization (ATRP), a technique, which allows the production of a variety of polymers [43]. This approach is utilized to polymerize functionalized methacrylate onto carbon-coated cobalt nanoparticles in order to achieve stable dispersions of MNPs [38]. These functionalized nanoparticles have an azide moiety, which may be further modified by “click”-reaction to include substrates that may be used for biomedical applications [38]. An additional biocompatible polymer is polyglycerol (PG). Polyglycerol has a chain structure similar to polyethylene glycol (PEG). The advantage of PG is its optimal hydrophilicity, stability and resistance to non-specific adsorption of proteins [44]. A simple one-step synthetic approach for PG is anionic ring opening polymerization, which results in a hyperbranched polymer. Biocompatibility of such PG was tested. Results revealed similar or even better behaviour of PG compared to PEG [45, 46]. Recently, hyperbranched PG was polymerized onto MNPs (Fe_2_O_3_). As a consequence, MNPs have become resistant to nonspecific adsorption of proteins [47]. Due to the simple synthesis process, the biocompatibility as well as the possibility for further functionalization, PG coating is a valuable alternative approach for the preparation of magnetic nanosensors for biomedical applications.

## Opportunities using magnetic nanosensors

The use of magnetic nanosensors in clinical applications will be discussed based on the two categories of intracorporeal (in vivo) and extracorporeal applications. This will be covered in the sections *Magnetic nanosensors as carrier system*, focusing on in vivo methods, and *Magnetic nanosensors as elimination system* for the extracorporeal removal of a molecular or cell entity from blood.

### Magnetic nanosensors as carrier system

#### Drug delivery

Magnetic nanoparticles are considered ideal candidates for drug delivery for several reasons. Their large surface-to volume ratio allows for a high loading with active substances. Moreover, these MNPs can be directed by a magnet and facilitate targeted delivery of drugs. Finally, stable dispersions and fast transportation in fluids can be realized due to the small size of the MNPs.

**Fig. 2:**
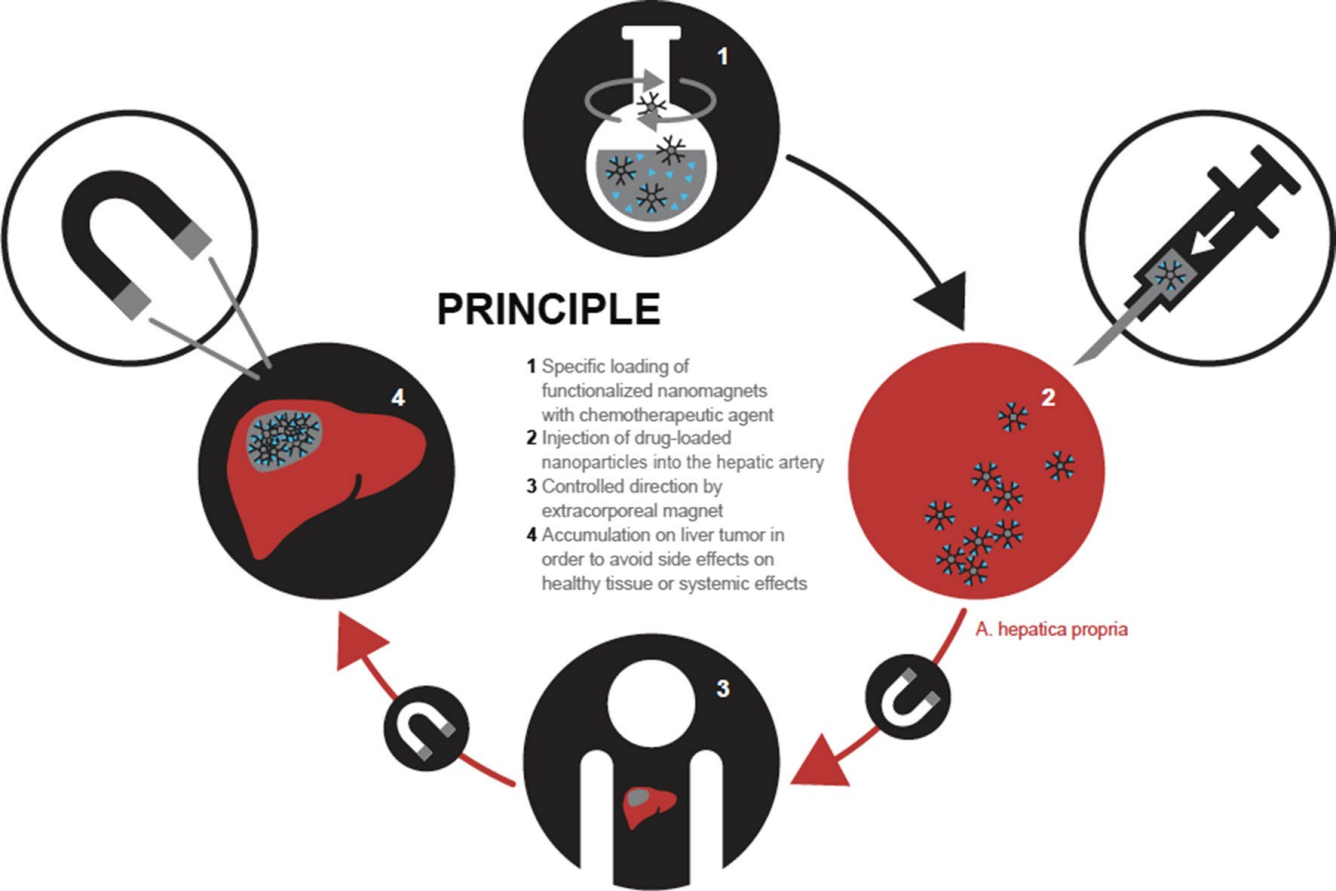
Magnetic nanosensors loaded with a chemotherapeutic to be injected intravenously and directed with a magnet towards the tumor tissue. In this specific example, the particle bound chemotherapeutic drug is introduced in the blood stream to treat a liver tumor. Preferred deposition of the drug in the liver through application of a local magnet improves the concentration of drug in the target organ and is expected to reduce side effects as less of the toxic drug reaches other organs

A subcategory for drug delivery is the group of chemotherapeutics. Classically, therapy with a chemotherapeutic drug is non-specific and the drug is applied systemically. Therefore, non-specific targeting of cells leads to many unwanted and sometimes severe side effects. Magnetic particles are engineered with a chemotherapeutic agent and designed to be target specific, reaching the area of the tumor cell with the help of a magnet (Fig. 2). Functionalized MNPs are internalized through caveolae structures or by endocytosis, a process, which is facilitated by specific receptors, [48]. Once in the cytoplasm, the drug is released performing the desired action in the target cells. Ideally, MNPs are then subsequently biodegraded [49]. To enhance the ability of MNPs to reach the targeted tumor cells, MNPs are often functionalized with antibodies in addition to their transporting drug. These antibodies support targeting of the tumor cell by specific binding and allowing for a precise treatment of the targeted tissue [50–52].

Similar to the loading of MNPs with chemotherapeutics, MNPs can be equipped with radioactive isotopes or radionuclides. The radionuclide-loaded MNPs are guided to the tumor cells and, upon internalization, kill then cancer cells through continuous irradiation [53–55]. Half-life times of the used radioactive isotopes have to be considered for the treatment to allow for efficient tumor cell killing, but at the same time not damaging normal tissue once the cancer cells have been eliminated.

Genetic disease treatment presents a further possible field of application for magnetic nanosensors. Classically, patients with a genetic disease, are treated with exogenous DNA to correct mutations, which are responsible for the disease. Also, antisense-RNA can be used to silence defective genes. However, with the current treatment methods different challenges are faced: (1) There is a clear lack of tissue specificity. (2) Transfection efficiency needs to be improved as introduction of the nucleic acids into cells is difficult using classical approaches [56]. (3) The life time of the DNA is very limited since it degrades fast. Therefore, MNPs may pose as a possible efficient transport system for gene therapy. The ability to target specific tissue and increasing transfection efficiency would overall augment gene transfer [57].

#### Contrast agent carrier

Another group of MNPs are magnetic nanosensors used as contrast agents in MRI to image specific organs. After injection, the magnetic nanosensors agglomerate in specific areas and—upon irradiation with radio waves—enhance the contrast and therefore increase image quality [58]. Such contrast agents are delivered as SPIONs, coated with hydrophilic polymers for stabilization in solution. Some SPION-based contrast agents are clinically approved and in use for liver, bowel and vasculature imaging [11, 59].

#### Hyperthermia

Similar to the use of functionalized MNPs as contrast agents is the technique of hyperthermia. A rise in tumor temperature makes cancer cells more susceptible to chemotherapy or radiation, and can directly cause cellular death. Therefore, MNPs as energy-transducing particles may be used locally to overheat tumor cells. This would be an attractive method for treatment of deep tissue seated tumors [60, 61].

### Magnetic nanosensors as elimination system

Blood purification describes the second category where magnetic nanosensors may be applied clinically, focusing on removal of non-cellular and cellular compounds. The challenge for magnetic nanosensors in blood purification lies in the functionalization of the MNPs with a suitable linking agent such as a metal coordinative ligand, a peptide or an antibody to bind solely the specific target [12, 62].

#### Removal of both low- and high-molecular weight blood compounds

**Fig. 3:**
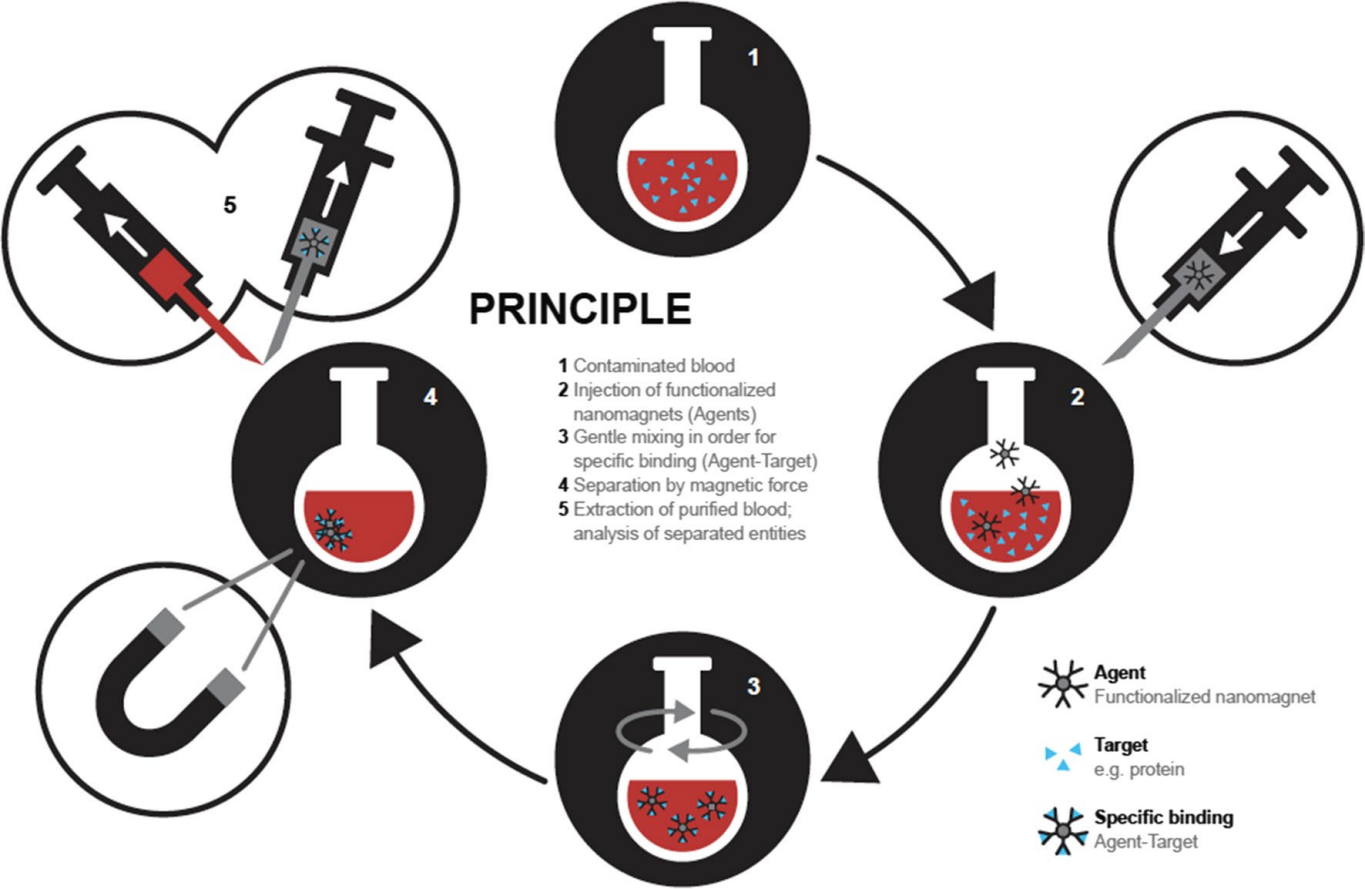
In an ex vivo approach the injected nanosensors are derivatized as to catch a contaminant of interest after mixing. Beside elimination of contaminants, a magnetic separation step also allows recollecting the injected agents, and separately removing the caught contaminants from the carrier, followed by an analysis of the desorbed contaminants. The possibility to sample contaminants in larger blood volumes but desorb the collected material into a small volume further permits up-concentration thus facilitating the detection of low concentration contaminants or biomarkers

**Fig. 4:**
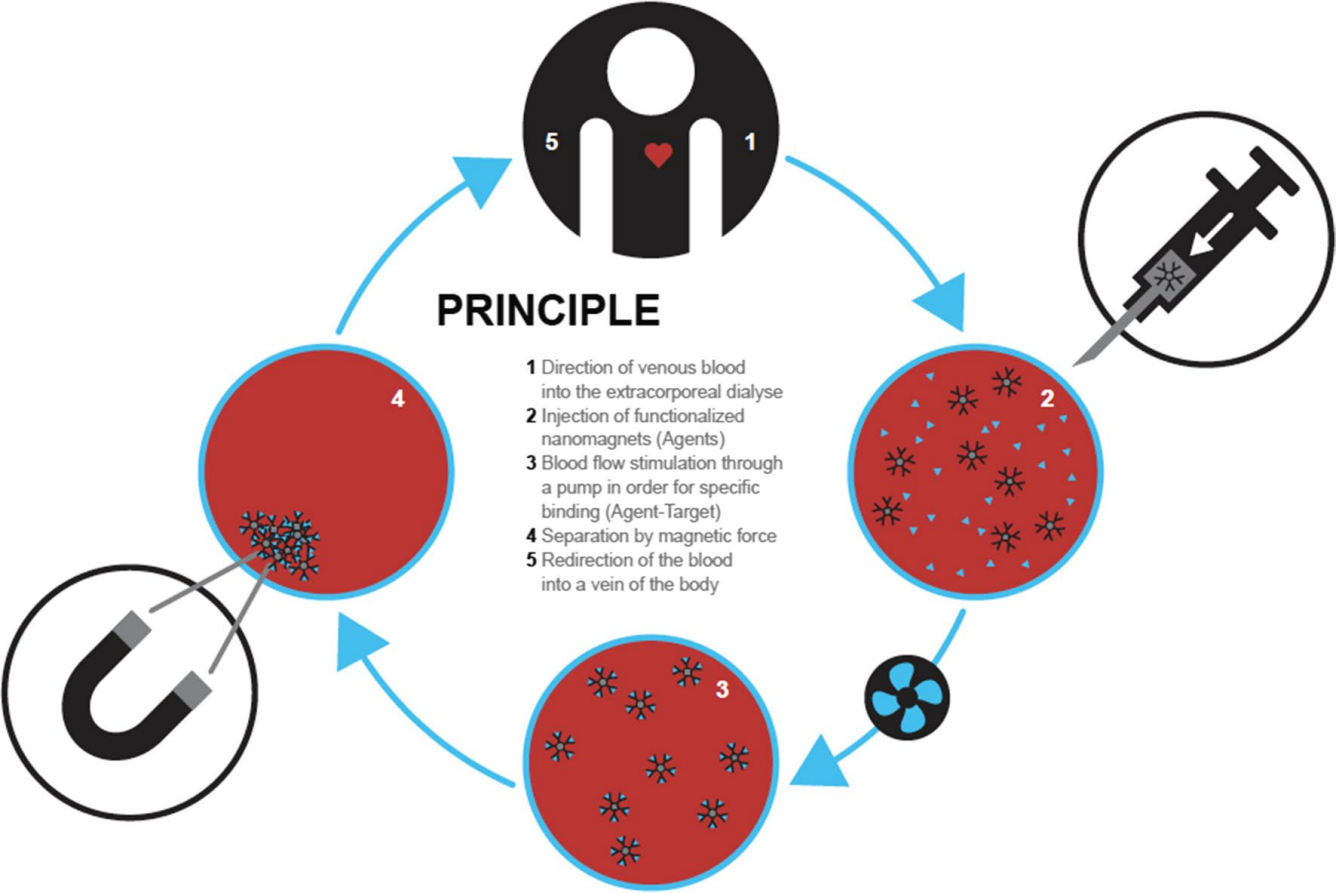
The therapeutic use of magnetic nanoparticles to remove significant parts of a blood contaminant requires injection and mixing of the magnetic particles during a prolonged time. After binding, the injected agent has to be removed in a magnetic separation step and ideally guarantees that essentially no particles are fed back into the patient’s body. In a typical clinical setup, a diagnostic step with detection of specific targets is expected to then lead to the treatment step using an adapted mix of magnetic agents

While current methods such as adsorption, filtration or dialysis clearly allow elimination of small molecules (i.e. potassium, urea, creatinine), harmful substances are often biomolecules of large size (i.e. antibodies, endotoxins, etc.). To remove complex-structured compounds, plasma exchange is mandatory with the disadvantage of loss of plasma [63]. As an alternative, filtration through antibody-coated columns is an option, but the nature of the compound has to be known with an according antibody being available [64]. Therefore, magnetic nanosensors are an interesting approach to reliably eliminate all kind of different compounds from the blood in an extracorporeal device approach (basic principle described in Fig. 3) (extracorporeal purification system described in Fig. 4).

Iron carbide nanoparticles have been functionalized in diverse ways to purify blood from metal ions, steroid drugs (i.e. digoxin known as an antiarrhythmic agent) as well as proteins (i.e. interleukins, IL, as inflammatory mediators). Successful elimination of lead, digoxin and IL-6 was achieved with an EDTA-like chelator as metal coordinating ligand, with an antibody such as digoxin-binding FAB fragments or with an anti-IL-6 antibody, respectively [65]. With the help of an extracorporeal blood purification system, these in vitro results were successfully reproduced in vivo in rats [66]. Particularly digoxin data were intriguing as they clearly showed a lowering of digoxin concentrations from a toxic to a therapeutic blood level within a short time.

When considering clinical scenarios, which would profit from blood purification using magnetic nanosensors, sepsis may be a main focus. Sepsis is a complex overwhelming response of the body to a systemic infection. It is potentially life-threatening as it often leads to organ failure and finally to death [67]. On one hand, the presence of extensively produced inflammatory mediators such as cytokines or complement products could be decreased in an extracorporeal blood purification approach with the help of magnetic nanosensors. It has been hypothesized that the removal of such entities would be highly beneficial [68] (Fig. 4). On the other hand, also toxins could be eliminated. Polymyxin B, an antibiotic, which binds and inactivates endotoxins, was used to functionalize magnetic nanoparticles. In a first approach, human blood contaminated with endotoxin, was successfully purified with these polymyxin B-coated MNPs (Fig. 3) [69]. Recently, SPIONs have been functionalized with peptides from binding motives of agglutinating salivary proteins acting as specific pathogen scavengers. These functionalized SPIONs were then utilized to bind endotoxin [70]. In another approach, the capturing of lipopolysaccharides (endotoxins) with functionalized MNPs was studied. The authors produced particles composed of iron oxide nanoparticles and macrophage membranes to capture lipopolysaccharides in vivo in rats [71]. These nanoparticles significantly attenuated systemic inflammation. At the same time, mortality of endotoxemic rats was decreased.

#### Removal of blood cells

Another area of interest in blood purification is the identification and/or removal of blood cells. This gives rise to the possibility of fast and efficient detection and treatment of diseases. A classical way of cell separation is based on cell size with the disadvantage, however, of not being able to separate cells of similar sizes. Also, when using the affinity cell separation technique, chemical or electrical properties may be similar in different cells. Magnetic separation based on the use of nanoparticles suffers from no such limitation as MNPs are coated with specific antibodies directed against a surface marker of the desired cells [72].

Isolation of hematopoietic stem cells from bone marrow or peripheral blood from a healthy donor presents a realistic future application for MNPs. Such donor cells are used to reintroduce functional stem cells in a recipient after chemotherapy for leukemia or lymphoma with killing of not only neoplastic, but also of growing cells by the cytotoxic agents. Although due to the low abundance of stem cells in bone marrow aspirate or peripheral blood, it is challenging to separate them, the use of magnetic nanosensors may present a suitable approach [73].

Another application for the removal of cells may be explored targeting circulating tumor cells (CTCs). These cells are thought to be a key factor in the process of metastasis [74]. Their presence in the blood indicates poor outcome [75, 76]. Elimination of CTCs via hemodialysis might enforce the suppression of further metastasis, thereby improving outcome.

Important to note when targeting blood purification with magnetic nanosensors is the need for a suitable device, which allows for efficient injection, mixing and removal of the magnetic nanosensors [77–81] (Fig. 4).

### Requirements for specific applications using magnetic nanosensors

Among magnetic nanosensors certain characteristics are shared for in vivo as well as extracorporeal applications. First and foremost, the particles need to be biocompatible. Therefore, they should refrain from non-specific adsorption as well as from agglomeration (unless desired in the target tissue). Second, it is a prerequisite that the particles are non-toxic. Not only cytotoxicity, but also tissue inflammation should be avoided. These are key parameters for the safe use of nanosensors, which are discussed later in the risk assessment part. Even magnetic nanosensors are used in a similar way in vivo and extracorporeally, different requirements need to be met for each application. Generally, the particles used for in vivo applications are smaller to account for faster transport and tissue penetration inside the body in comparison to extracorporeal separation applications. Additionally, for in vivo applications the nanoparticles need to be either small enough to be filtered by kidneys or big enough to be eliminated by liver and spleen or they are biodegradable. All these characteristics decrease the risk for accumulation in the body.

Specific requirements for the synthesis of magnetic nanosensors depending on the purpose of the application are summarized in Table 1. Blood purification was taken as a single application since the requirements needed are similar for separation of small moieties as well as cells.

**Table 1:** Requirements for in vivo and extracorporeal applications using magnetic nanosensors

Application	Requirements	References
Drug delivery	Stability of drug-nanoparticle composite to guarantee no premature release Composite optimally entirely biodegradable High magnetization for precise and efficient magnetic transport	[8, 82]
Contrast agents	High magnetization desired to achieve good contrast Ability to strongly shorten relaxation times in tissue	[83, 84]
Hyperthermia	Specificity needed towards target tissue and ability to penetrate cells Magnetic anisotropy (shape, crystallinity, surface properties) and size/size-distribution influence heating efficiency 15–20 nm core size optimal with low dispersity to achieve high heating efficiency	[61, 85–87]
Blood purification	Good dispersions for optimal binding speed to target High magnetization or larger particles (for SPIONs) for a later efficient separation Stable coating, non-interactive with other blood species High specificity needed for removal of non-abundant moieties (e.g. CTC)	[88–90]

## Risk assessment of MNPs for clinical applications

When considering in vivo as well as extracorporeal MNP applications safety aspects are of utmost importance. In general, since the discovery of the nanoscale, nanoparticles opened up new fields of research and subsequently various applications of nanoparticles were found. Due to their small size, nanoparticles were initially believed to have little to no influence on living organisms. Only later, when researchers started broader investigations regarding possible risks of nanoparticles, adverse effects were discovered.

In vitro assessment of nanoparticles concerning toxicity is undertaken in the same way as the assessment of chemical compounds in solution [91]. This is problematic due to the fact, that nanoparticles do not have the same properties as chemical compounds in solution with a possible different behaviour. Therefore, it is important to formulate protocols tailored to the risk assessment of nanoparticles including all possible aspects of harm MNPs could present with [92]. Over the last few years the Center for Drug Evaluation and Research within the FDA has supported and performed many studies concerning the regulatory aspect in risk assessment of drug-containing nanomaterials [93]. Generally, evaluations of such nanomaterials are performed on a case-to-case basis, and protocols are then established and implemented for the risk evaluation of nanoparticles in a more universal way. This clearly poses a challenge as the variety of nanoparticles produced is ever expanding and new possible nanoparticles with different shapes, compositions and surface functionalizations are produced with unforeseeable effects towards organisms.

Various studies exist assessing possible toxic effects of MNPs in a living organism. Surface characteristics determine distribution within the body, whereas size, dose and entry point of nanoparticles are important as well. In general, inflammation may be triggered through stimulation of effector cells, producing proinflammatory mediators, whereas the proinflammatory effect seems to be surface-dependant [94]. When MNPs remain in the tissue over time, chronic inflammation may be another consequence leading to fibrosis of the affected organ [95, 96]. Finally, MNPs may evoke damage, which triggers the development of cancer [97]. This is of particular concern as long-term studies are still missing.

Nanoparticles, once present in the body, may target various systems. There are effects of nanoparticles found on the circulatory system, where nanoparticles indirectly influence for example blood pressure [98]. Important to note when looking at the circulatory system is the fact that nanoparticles are engineered to influence the coagulation system of the blood [99]. At the same time, MNPs, designed for any other indication, may evoke an unwanted pro- or anticoagulant effect in the blood [100].

A recent study mimicked the clinical scenario with ferromagnetic iron carbide nanoparticles used for blood purification methods, which theoretically escaped magnetic separation and entered the blood system of mice [101]. These particles mainly accumulated in the lungs, liver and spleen [101]. Although, MNPs were still present in the organs after a year, they were well tolerated and no significant immunological response was detected over time [101].

The reproductive system is another target of nanoparticles with possible detrimental effects. Upon in vivo applications, nanoparticles may accumulate in reproductive organs [102, 103], where they have direct effects on germ cells with reduced cell count or activity in both, female and male germ cells [104, 105]. Furthermore, nanoparticles are able to alter or damage DNA in cells, which would be especially problematic in germ cells [106]. In a recent in vitro study, uptake of coated SPIONs in granulosa cells was tested. It was found that depending on the coating, no or only low uptake and toxicity of SPIONs was observed [107].

All these various possible effects that certain nanoparticles may or may not exhibit display the challenge in the risk assessment for nanoparticles in medical applications. Regulatory and toxicology studies have to address the fate of such nanosensors. Therefore, available magnetic nanomaterials for in vivo applications become limited. Up to now only SPIONs, due to the biodegradability of iron oxide, are clinically used. Non-biodegradable MNPs, even though they may be biocompatible, pose a challenge as accumulation and therefore unknown and detrimental effects in tissue are possible. Mechanisms are needed, which allow total excretion of such non-biodegradable MNPs. To the best of our knowledge, this is so far not achieved.

In nanosensor systems where the particles eliminate compounds from the blood, ideally all MNPs are removed from the blood with a strong magnet before the blood is redirected into the body. Therefore, biodegradability and excretion is not a relevant aspect. This alters the question relevance for regulatory approval. Nevertheless, biocompatibility in blood, dispersability and the subsequent removal of the magnetic nanosensors are to be considered. Reintroduction of the blood has to be performed excluding even traces of nanosensors. Elsewise, they will be introduced into the bloodstream of the patient and this may have harmful consequences [108].

Recently, a method for detection of trace amounts of MNPs in complex fluids was published [89]. The authors used a magnetometric sensor, which detects low magnetic fluctuations to determine the presence of iron and cobalt MNPs under flowing conditions. The advantage of this method is not only the very low detection limit, but also the sample is not destroyed during analysis, which enables on-line detection. It is believed that this method may be used in combination with a blood purification device to reliably proof that no particles are introduced into the patient. Additionally, when combining this method with protocols for safe handling of MNPs, the acceptance of using MNPs for blood purification may be further strengthened.

## Future perspectives

The use of magnetic nanosensors in clinical applications has seen great advances over the last few years. Magnetic nanosensors for MRI are readily used as contrast agents in clinical applications [6, 59]. Additionally, with the possibility to combine diagnostics and simultaneous therapy, so called theranostics, a new class of functionalized MNPs may be used in the future for clinical applications [109, 110]. Concerning gene therapy, the main challenge still lies in the production or functionalization of suitable MNPs and risk assessment thereof.

In extracorporeal applications for magnetic nanosensors decent progress has been made. With CliniMACS^®^, a method to separate T-Cells from blood, a first FDA-approved clinical application for magnetic nanosensors has been established [111].

When looking towards the treatment of sepsis, a lot of research for the application of magnetic nanosensors is currently ongoing, academic and industrial wise [112]. Several improvements still have to be done. At one point, the device for the removal of MNPs should match the required flow rates and separation efficiencies in order to avoid any magnetic nanosensors to be directed into the blood stream. Concerning the functionalization of the magnetic nanosensors to apply in blood purification to treat sepsis, particles have to be developed, which remove a wide range of sepsis causing pathogens and additionally remain inert to any other cell or molecular entities in blood. This could be bypassed by creating mixtures of different magnetic nanosensors, each one with a binding site functionalized to remove a specific sepsis-causing pathogen or to eliminate an inflammatory mediator or complement products.

Even though the progress in possible clinical applications is visible, risk considerations may not be left out. A magnetic nanosensor needs to be safe for handling and treatment. There are still possible unclear variables towards the safety of magnetic nanosensors. Not to forget is the fact that long-term studies with magnetic nanosensors are not yet available to address and define possible long-term effects of such nanoparticles.

To sum up, magnetic nanosensors towards medical applications, show great promise as novel medication-, diagnostic- and separation tools. The variety of already approved magnetic nanosensors as contrast agents in MRI boost the confidence that magnetic nanosensors may also be reliably applied in vivo for targeted drug delivery. For extracorporeal applications, the treatment of sepsis with magnetic nanosensors shows great promise should it become commercially available.

## Data Availability

References were found through PubMed search.

## Abbreviations

MNP: magnetic nanoparticle
MRI: magnetic resonance imaging
SPION: super paramagnetic iron oxide nanoparticle
ATRP: atomic transfer radical polymerization
PEG: polyethylene glycol
PG: polyglycerol
CTC: circulating tumor cells
FDA: United States Food and Drug Administration
